# Composite-Structured Anti-Resonant Fiber with High Temperature Sensitivity for Cancer Cell Detection

**DOI:** 10.3390/s26123670

**Published:** 2026-06-09

**Authors:** Ruifan Wu, Qiming Wang, Yongqi Gai, Xiaolan Zhang, Xinru Shan, Danping Jia

**Affiliations:** School of Information Science and Engineering, Shenyang University of Technology, No. 111, Shenliao West Road, Economic & Technological Development Zone, Shenyang 110870, China; wuruifan@smail.sut.edu.cn (R.W.);

**Keywords:** anti-resonant fiber, SPR effect, temperature sensing, cancer cell detection

## Abstract

This study proposes a novel anti-resonant fiber sensing structure based on a composite “egg-shaped” configuration with surface plasmon resonance (SPR) effect. By designing a novel anti-resonant structure consisting of a semicircle and a semi-ellipse and coating its inner surface with a gold film, the optimal structural parameters are determined through three sets of simulation experiments using temperature sensitivity as the criterion. The optimal sensing structure was applied to the simulated detection and analysis of cancer cells, aiming to provide value and reference for the application of high-sensitivity optical fiber sensor in the field of cancer cell detection. Simulation results show that the proposed sensing structure achieves a maximum temperature sensitivity (TS) of 3.86 nm/°C. For the detection of six different types of cancer cells, the maximum wavelength sensitivity (WS), optimal resolution (R), maximum figure of merit (FOM), maximum signal-to-noise ratio (SNR), and best limit of detection (LOD) reach 12,142.86 nm/RIU, 8.24 × 10^−6^, 3035.72 RIU^−1^, 65.50, and 0.94 nm, respectively. Owing to its unique detection mechanism, the proposed sensing structure exhibits label-free characteristics and demonstrates balanced and excellent performance across all metrics for both temperature and cancer cell detection, showing broad application prospects and great potential in the fields of environmental monitoring and medical prevention and treatment.

## 1. Introduction

With the rapid development and expanding applications of photonic crystal fibers (PCFs) in recent years, their potential in the field of high-sensitivity and high-resolution sensing has become increasingly prominent [1]. Among them, anti-resonant fibers (ARF), as a breakthrough in the new category of PCFs, rely on the anti-resonant reflecting optical waveguide (ARROW) principle [2] and possess superior optical properties such as ultra-low transmission loss [3], high transmission bandwidth [4], low nonlinearity [5], and ease of use [6], thus attracting widespread attention. Meanwhile, fiber-optic sensors based on the SPR effect have been widely applied in fields such as biomedicine [7] and ecological environment [8] due to their advantages of high sensitivity, label-free detection, and real-time monitoring. Therefore, the novel ARF based on the SPR effect will offer unique advantages and hold significant importance in the fields of medical prevention/treatment and environmental monitoring.

Temperature is a core parameter affecting life, health, and the environment [9], and its high precision and high sensitivity are in urgent demand in cutting-edge fields [10]. Meanwhile, cancer is a disease characterized by uncontrolled cell proliferation driven by genetic mutations. As the leading cause of disease-related death, its incidence and mortality rates remain persistently high. Therefore, early prevention and detection have become a top priority in the long-term task of biomedical research. In recent years, fiber-optic sensing structures based on the SPR effect have been continuously developed for temperature and cancer cell detection. In terms of temperature sensing, Zhang et al. proposed and demonstrated a hybrid fiber sensor integrating a multimode interference (MMI) structure with an SPR structure, achieving a TS of 37.9 pm/°C [11]. Zhang et al. also proposed an SPR sensor based on a fan-shaped microstructured optical fiber (MOF), which exhibited a TS of 2.932 nm/°C in the range of 30 °C to 40 °C [12]. Wang et al. proposed a high-sensitivity SPR sensor based on a seven-core photonic crystal fiber (SCPCF), achieving a TS of −2.59 nm/°C [13]. Su et al. proposed an all-fiber S-shaped structured fiber SPR sensor, with a maximum TS of −0.713 nm/°C [14]. Du et al., for the first time, proposed an S-shaped fiber SPR sensor structure, achieving a TS of −1.6107 nm/°C in the range of 20 °C to 65 °C [15]. An et al. proposed a dual-parameter sensor based on a hybrid sensing mechanism of SPR and mode resonance coupling using a D-shaped hole-assisted dual-core fiber (HADCF), achieving a TS of 2.86 nm/°C [16]. In terms of cancer cell detection, Ibrahimi, Khalid Mohd et al. proposed a novel SPR biosensor based on a TiO_2_/Au hybrid layer, achieving a maximum WS of 4285.71 nm/RIU [17]. Vijay Shanker Chaudhary et al. proposed a SPR refractive index sensor based on a PCF coated with gold (Au) and titanium dioxide (TiO_2_), achieving a maximum WS of 10,714.28 nm/RIU [18]. Md. Ranju Sardar et al. designed a novel PCF-SPR sensor, achieving a maximum WS of 7143 nm/RIU [19]. B. Nagavel et al. proposed a PCF-based SPR biosensor with dual-core and dual-side surface detection capability, achieving a maximum WS of 5714.28 nm/RIU [20]. Mohammed F. Majeed et al. proposed an SPR biosensor based on a novel dual-core PCF, achieving a maximum WS of 11,429 nm/RIU [21]. Khalid Mohd Ibrahimi et al. proposed a dual-core C-grooved high-sensitivity PCF SPR biosensor based on a graphene gold hybrid structure, achieving a maximum WS of 2142.86 nm/RIU [22]. To date, few reports have been published on novel high sensitivity anti resonant fiber structures for medical detection.

This paper designs a novel anti-resonant fiber with an “egg shaped” structure based on SPR effect. A dual cavity structure consisting of a semicircle and a semi ellipse is designed and coated with a gold film on the inner surface. Using optimal TS as criterion, three sets of experiments are conducted to determine optimal structural parameters, namely elliptical curvature, cladding tube thickness, and gold (Au) film thickness. The resulting optimal structure is then applied to detect and distinguish six types of cancer cells: blood cancer (Jurkat), skin cancer (Basal), cervical cancer (HeLa), two types of breast cancer (MCF-7 and MDA-MB-231), and adrenal gland cancer (PC12). Simulation results show that this study achieves high performance indicators for both temperature and cancer cell detection, enabling sensitive detection of temperature changes and discrimination between normal and cancer cells. This study holds promising prospects and translational application potential in the fields of temperature monitoring and biosensing for cancer cell detection.

## 2. Research and Analysis

### 2.1. Liquid Filling of the Fiber Core

In the study of hollow-core anti-resonant fibers, it is often necessary to fill the fiber core with a liquid to achieve better sensing performance [23]. When the optimal structural performance is determined through temperature variation, the refractive index (RI) of the liquid core needs to change accordingly. Anhydrous ethanol has a moderate RI, which facilitates suitable mode coupling conditions in the anti-resonant structure, and it has a good application foundation in optical fiber sensing research, making it easy to implement and apply. Moreover, because the thermal expansion coefficients of both silica and the gold film are very small, the deformation caused by temperature changes can be neglected [24]. Therefore, anhydrous ethanol, which is stable and convenient, is selected as the filling liquid in this work. The RI of anhydrous ethanol as a function of temperature is shown in Table 1 [25].

**Figure 1:**
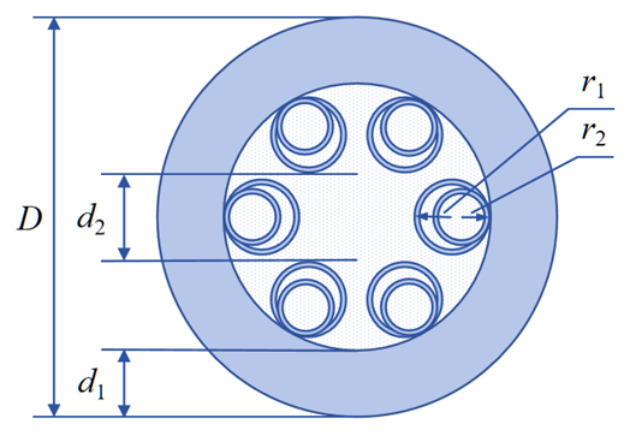
The underlying configuration.

The basic configuration adopted in this study is shown in Figure 1. Based on a circular nested hollow-core structure, anhydrous ethanol is used as the liquid core to fill the core region. The outer cladding has an outer diameter *D* = 260 μm and a thickness *d*_1_ = 30 μm. The fiber core diameter is *d*_2_ = 100 μm. The outer radius of the cladding tube is *r*_1_ = 25 μm, and the inner radius is *r*_2_ = 20 μm. The software used for the simulation experiments was COMSOL Multiphysics 6.2. In the simulation experiments, the physics field was set to electromagnetic waves, frequency domain (EWFD). A silica outer cladding layer with a thickness of *d*_1_ was configured as a perfectly matched layer (PML) to absorb the leaked optical field. The finite element method (FEM) was employed for the analysis. This method partitions different structures into meshes of varying shapes and densities according to actual requirements, and is capable of analyzing structures with complex geometries and handling arbitrary boundary conditions, thereby yielding the corresponding solutions.

### 2.2. Egg-Shaped Cladding Tube Structure

**Figure 2:**
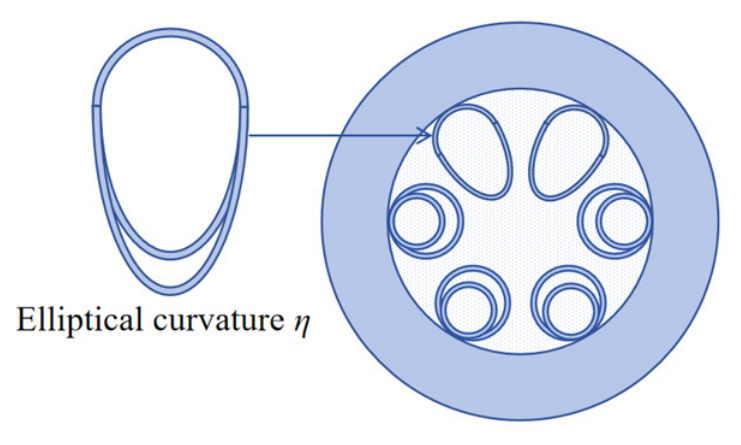
Different ellipse curvature structures.

The fundamental configuration of the anti-resonant fiber in this study represents a new breakthrough in photonic crystal fibers, relying on the unique anti-resonant reflecting optical waveguide (ARROW) principle. As light propagates within the fiber core, when the phase difference satisfies the resonance condition, the light leaks strongly, forming high-loss regions. Conversely, the capillary walls firmly confine the light beam within the air core, enabling low-loss transmission and forming low-loss regions. This mechanism provides the guarantee for ultra-high-sensitivity sensing and constitutes an advantage over conventional photonic crystal fibers. Meanwhile, the proposed anti-resonant fiber with a semi-circular and semi-elliptical “egg-shaped” composite structure is illustrated in Figure 2. The cladding of this liquid-core anti-resonant fiber consists of four circular nested anti-resonant units and two semi-circular and semi-elliptical composite structures. This “egg-shaped” anti-resonant structure brings the core optical field closer to the cladding on the elliptical side. The sharp curved surface of the elliptical cladding tube pushes the internal optical field in the core outward toward the metal film subsequently coated inside the composite structure, while also providing a wider range of incident angles for the optical field. Meanwhile, the curvature of the circular side expands the range over which the SPR effect is generated as much as possible, thereby achieving a more efficient SPR effect at the “egg-shaped” composite structure, which further enhances the sensitivity. Conventional circular and elliptical structures tend to generate the SPR effect locally at the tip, resulting in a relatively small effective area in some cases and a less pronounced effect. They may also cause the cladding tubes to be positioned too close to the lateral cladding tubes, leading to extra optical field coupling through the gaps rather than the SPR effect. Such coupling, in certain circumstances, can disrupt the overall anti-resonant effect and consequently compromise the sensing performance.

Since the basic principle of anti-resonant fiber sensing is that light propagates through reflection within the internal resonant cavity structure, it is inevitable that some light leaks through the glass wall. The resulting loss is known as confinement loss (CL), also referred to as leakage loss. The confinement loss is calculated from the imaginary part of the effective mode refractive index, *Im*(*n_eff_*), as shown in Equation (1) [26].

(1) $$CL=\frac{20}{ln10}\cdot \frac{2\pi }{\lambda }Im(n_{eff})\approx 8.686\cdot \frac{2\pi }{\lambda }Im(n_{eff})\;(dB/m)$$

**Figure 3:**
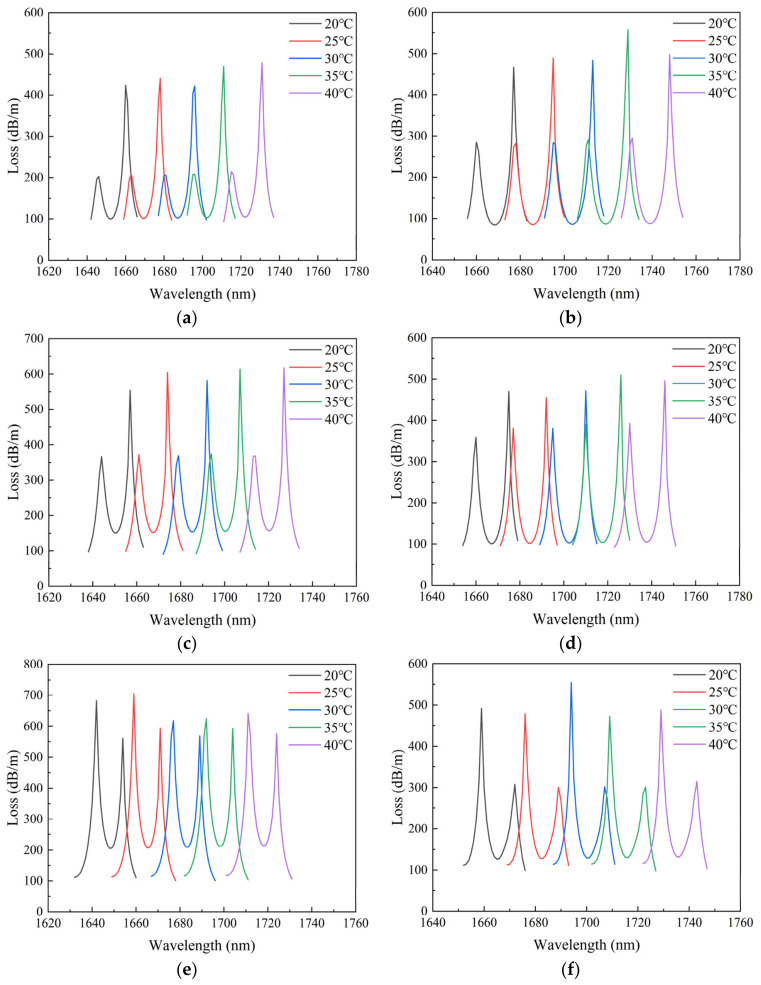
Loss peaks for different ellipse curvatures: (**a**) 26:48 X-polarization; (**b**) 26:48 Y-polarization; (**c**) 26:50 X-polarization; (**d**) 26:50 Y-polarization; (**e**) 26:52 X-polarization; (**f**) 26:52 Y-polarization.

Here, the curvature of the semi-elliptical cladding tube is defined as *η* = *d_minor_*/*d_major_*, where the radius of the semicircle is equal to the minor semi-axis *d_minor_* of the semi-ellipse. For ease of calculation and comparative analysis, the minor semi-axis *d_minor_* is kept constant in this study, while the length of the major semi-axis *d_major_* is varied. The cladding tube thickness is tentatively set to 0.9 μm, the gold film thickness to 0.1 μm, and all other basic parameters are kept unchanged. Simulations are performed with a step size of 1 nm to obtain the variation in the loss peak with changing elliptical curvature, as shown in Figure 3.

**Figure 4:**
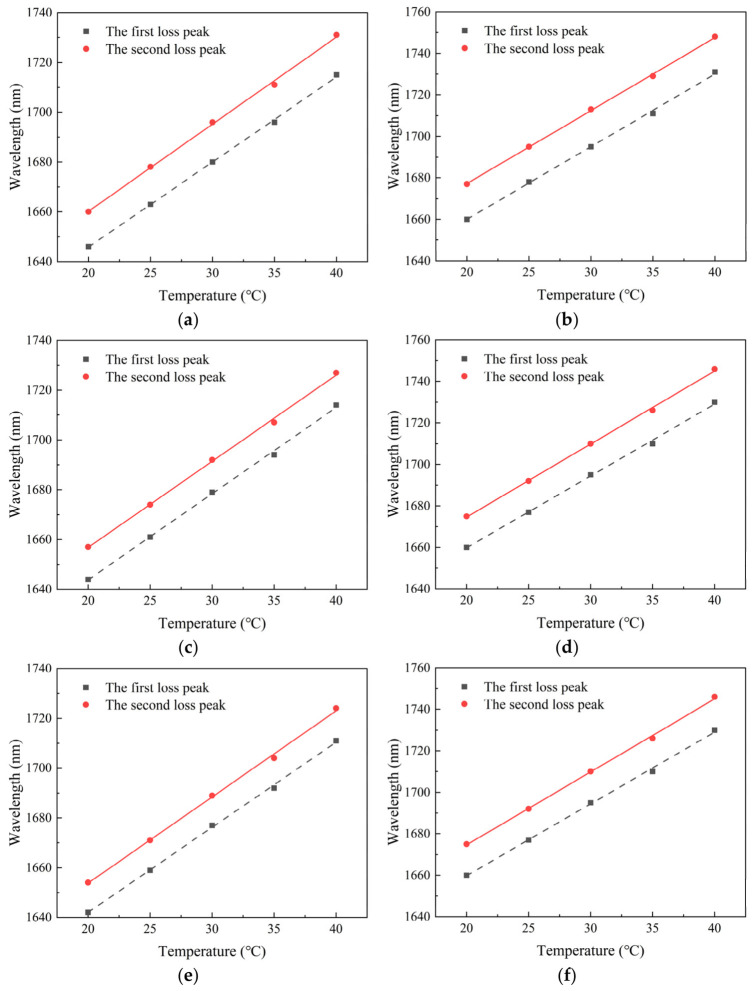
Linear fitting of different ellipse curvatures: (**a**) 26:48 X-polarization; (**b**) 26:48 Y-polarization; (**c**) 26:50 X-polarization; (**d**) 26:50 Y-polarization; (**e**) 26:52 X-polarization; (**f**) 26:52 Y-polarization.

It can be seen from the curves that as the elliptical curvature increases, the loss peaks for both X- and Y-polarizations exhibit a clear red shift, indicating improved performance. To more accurately evaluate the influence of the elliptical curvature variation on the model, the loss peaks corresponding to different elliptical curvatures for X- and Y-polarizations are linearly fitted separately, and the resulting fitted line graph is shown in Figure 4.

The TS is determined by the ratio of the shift in peak wavelength Δ*λ_peak_* to the temperature change Δ*T* [27], as shown in Equation (2). The calculated TS values are presented in Table 2.

(2) $$S_{T}=\frac{\Delta \lambda_{peak}}{\Delta T}\;(\mathrm{nm}/{}^{\circ}C)$$

It can be seen from Table 2 that as the elliptical curvature increases, the TS continuously improves, reaching a maximum of 3.52 nm/°C with an R^2^ of 0.9995. The results of this set of simulation experiments indicate that the TS performance is highest and best when the curvature *η* = 26:48. However, merely changing the shape of the cladding tube cannot achieve a significant improvement in TS, and further design based on this structure is still required.

### 2.3. Cladding Tube Wall Thickness

**Figure 5:**
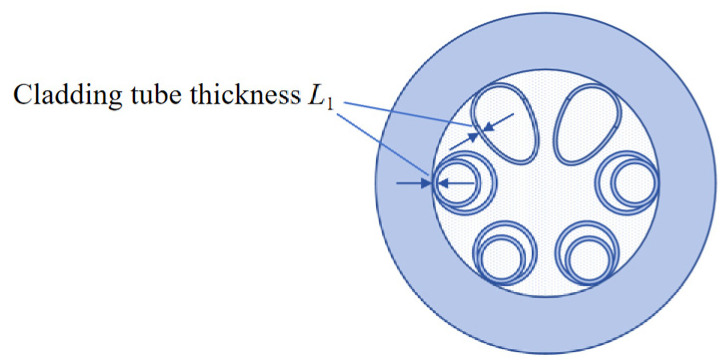
Different cladding tube wall thickness.

After determining the elliptical curvature, the cladding tube wall thickness was investigated to further improve the TS. Therefore, in this section, experiments were conducted for different cladding tube wall thicknesses *L*_1_, and the structural schematic is shown in Figure 5.

**Figure 6:**
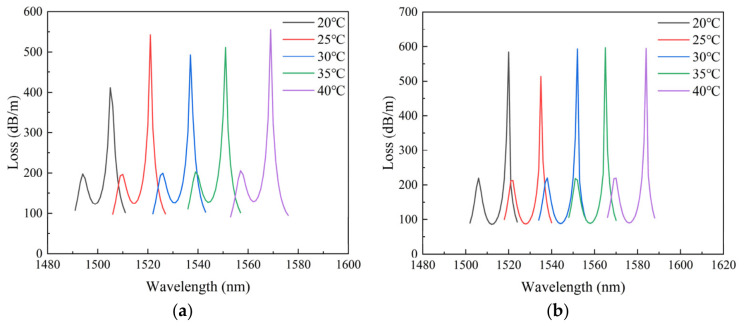

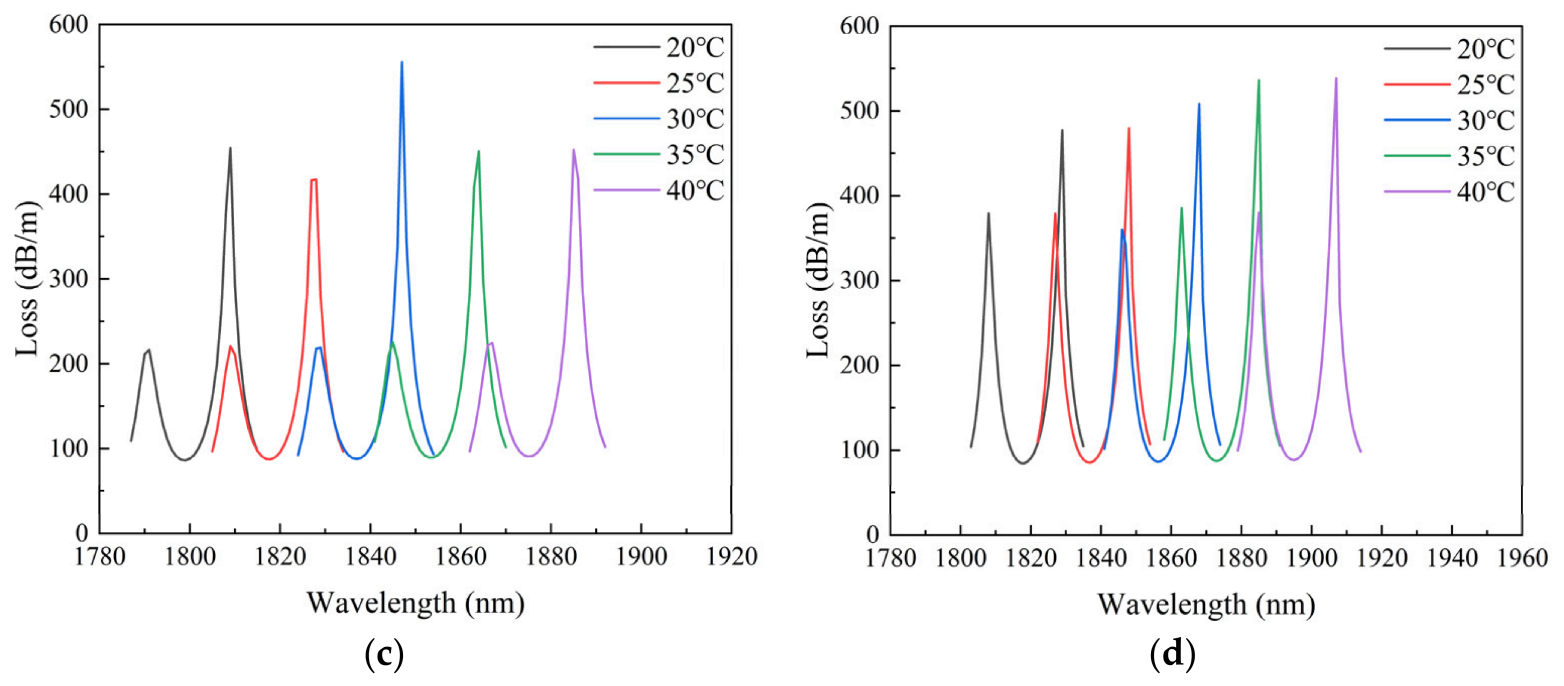
Loss peaks for different cladding tube wall thicknesses: (**a**) 0.8 μm X-polarization; (**b**) 0.8 μm Y-polarization; (**c**) 1.0 μm X-polarization; (**d**) 1.0 μm Y-polarization.

At this point, with all other basic parameters kept unchanged, simulations were performed with a step size of 1 nm to obtain the variation in the loss peak as the cladding tube thickness changes, as shown in Figure 6.

**Figure 7:**
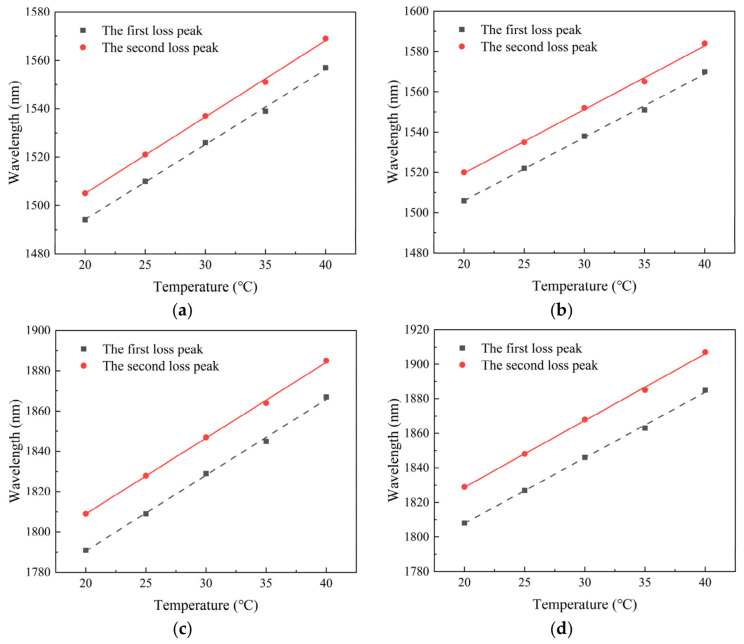
Linear fitting of different cladding tube wall thicknesses: (**a**) 0.8 μm X-polarization; (**b**) 0.8 μm Y-polarization; (**c**) 1.0 μm X-polarization; (**d**) 1.0 μm Y-polarization.

It can be seen from the curves that as the cladding tube thickness increases, the loss peak exhibits a clear red shift, indicating improved performance. The loss peaks were linearly fitted, and the resulting fitting plot is shown in Figure 7.

The calculated TS values are presented in Table 3.

It can be seen from Table 3 that, as the cladding tube wall thickness increases, the TS improves significantly. However, when a thickness of 1.1 μm was further selected for simulation, the wavelength band exceeded 2000 nm, making the light source difficult to obtain and the sensor less practical. In summary, the results of this set of simulation experiments indicate that when the cladding tube thickness is 1.0 μm, the highest TS reaches 3.86 nm/°C, with an R^2^ of 0.9989.

### 2.4. The Thickness of the Coated Gold Film

**Figure 8:**
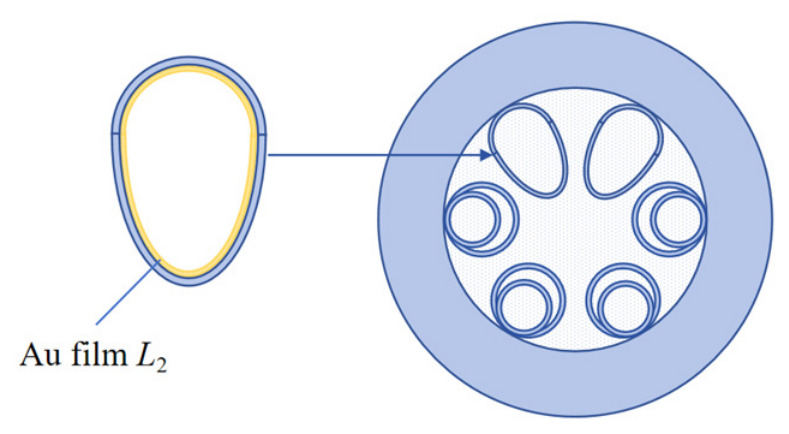
Different Au film thicknesses.

**Figure 9:**
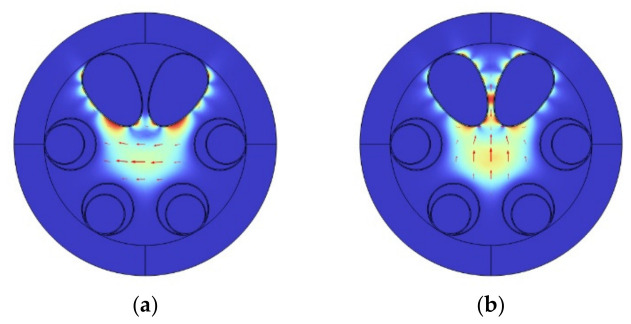
The cladding tube is coated with the Au film: (**a**) X-polarization; (**b**) Y-polarization.

From the two sets of simulation experiments on the elliptical curvature and the cladding tube wall thickness, it can be seen that the sensing performance can be improved by optimizing the structural parameters. In the composite structure, the SPR effect can also be excited by coating a metal film, further enhancing the TS. Among commonly used metal coating materials, gold exhibits a large complex RI and significant absorption characteristics in the visible wavelength range, which is favorable for exciting a pronounced SPR effect. Moreover, the deposition process for this coating material is well established, making it easy to prepare and implement. Therefore, gold is selected as the coating material in this study, and experiments are carried out for different gold film thicknesses *L*_2_. A schematic cross-sectional view of the structure after gold film coating is shown in Figure 8. When the phase difference satisfies the resonance condition, the incident light in the core couples with the free electron oscillations on the metal surface, exciting a surface plasmon polariton (SPP) mode at the interface between the cladding tube of the “egg-shaped” composite structure and the gold film. In this anti-resonant SPR fiber sensing structure, the SPP mode typically exists as a lossy mode. Upon coupling with the fundamental core mode, the confinement loss increases sharply, resulting in the appearance of an energy loss peak at a specific wavelength. The resonance profile displays a distinct and pronounced feature in which the core mode energy is significantly transferred to the metal film surface and becomes locally enhanced. In addition, in-plane arrows are added to illustrate the modal field polarization distributions of the sensing structure in the X and Y directions, as shown in Figure 9.

**Figure 10:**
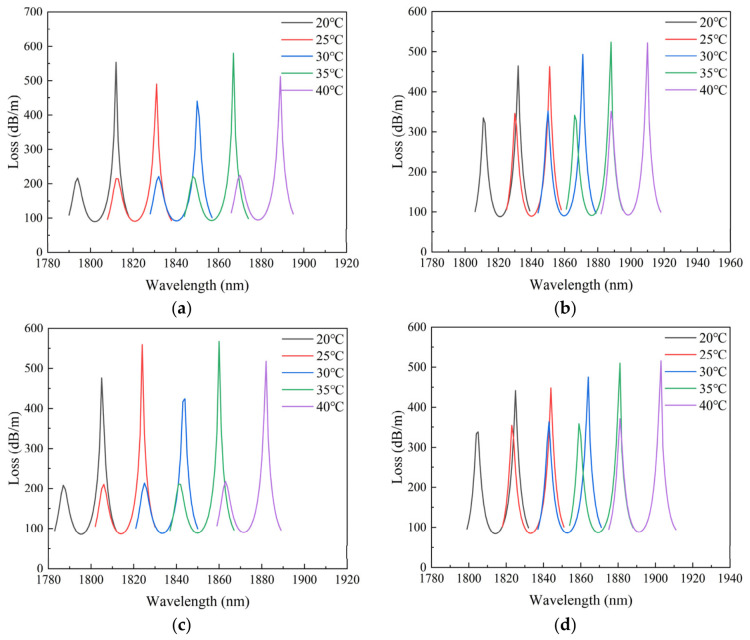
Loss peaks for different Au film thicknesses: (**a**) 0.05 μm X-polarization; (**b**) 0.05 μm Y-polarization; (**c**) 0.15 μm X-polarization; (**d**) 0.15 μm Y-polarization.

At this point, based on the optimal parameters obtained from the above two sets of simulation experiments and with all other basic parameters kept unchanged, simulations were performed with a step size of 1 nm to obtain the variation in the loss peak as the thickness of the coated gold film changes, as shown in Figure 10.

**Figure 11:**
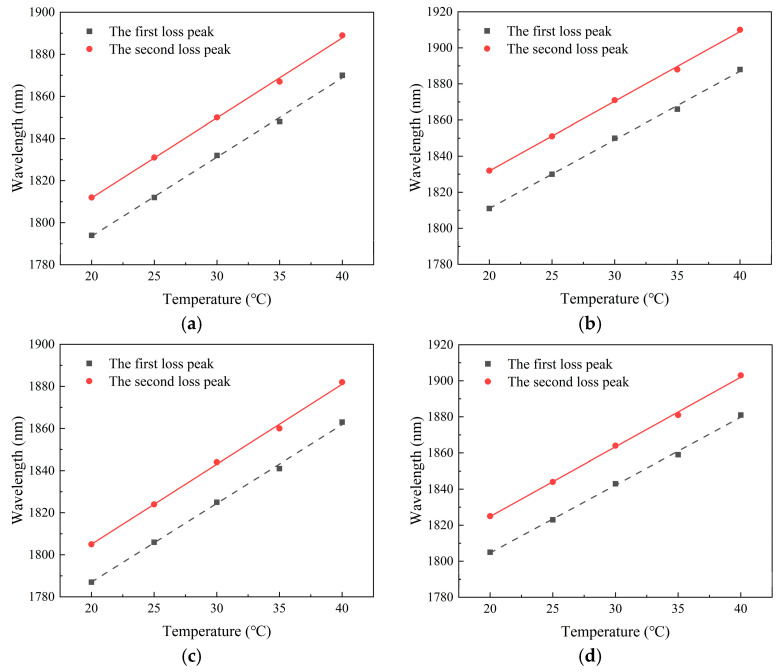
Linear fitting of different coating thicknesses: (**a**) 0.05 μm X-polarization; (**b**) 0.05 μm Y-polarization; (**c**) 0.15 μm X-polarization; (**d**) 0.15 μm Y-polarization.

It can be seen from the curves that as the gold film thickness increases, the loss peak exhibits a red shift, indicating improved performance. Linear fitting was performed again, and the resulting fitting plot is shown in Figure 11.

The calculated TS values are presented in Table 4.

It can be seen from Table 4 that as the gold film thickness decreases, the loss peak exhibits a red shift and the TS improves. However, when the gold film thickness was further reduced to investigate its effect on the TS, the results became erratic, indicating that the coated film should not be too thin.

In the fabrication of the anti-resonant fiber structure, the capillary stack-and-draw method can be employed to achieve precise alignment of capillaries with different wall thicknesses. Mahdiraji et al. have verified the process feasibility of this method in detail through experimental studies, demonstrating its capability to fabricate various types of photonic crystal fibers [28]. Alternatively, the extrusion method can be adopted. Cordeiro et al. demonstrated a technical route for single-step fabrication of complex microstructured optical fibers using a low-cost extruder, and successfully produced a hollow-core fiber through a 3D-printed nozzle [29]. Furthermore, for the complex “egg-shaped” structure inside the anti-resonant fiber, the 3D printing method possesses the capability to fabricate intricate air-hole shapes, such as rectangular, elliptical, and other non-circular cross-sections [30]. Once the internal microstructure is fabricated, a uniform nanoscale gold film coating can be deposited on the inner walls of the structure using the chemical deposition method. Considering the influence of fabrication deviations on fiber performance, Chen et al. conducted a systematic numerical study on the fabrication tolerances in inhibited-coupling guiding hollow-core fibers, pointing out that although precise control of the drawing parameters during fiber production is challenging, it is considered to be technically feasible [31]. Therefore, the sensitivity and tolerance issues arising from the minor deviations between the actual fabrication and the ideal model of the “egg-shaped” cladding symmetric structure adopted in this study can be addressed through precise control of the fabrication process. Moreover, with the rapid development of hollow-core fiber manufacturing technologies, new methods have emerged, such as the “stack, seal, evacuate, and draw” technique, which enables fibers to be drawn directly from a stacked-sleeve preform. By combining novel fabrication processes with independent control of each glass capillary during pressurization, a feasible approach for fabricating hollow-core photonic crystal fibers with a nearly perfectly symmetric structure has become available [32]. In this way, the influence of gold film thickness fluctuations and structural geometric deviations on the performance can be minimized as much as possible. Therefore, by leveraging existing mature microstructured optical fiber fabrication technologies, such as the capillary stack-and-draw method, the extrusion method, 3D printing, and chemical deposition coating techniques, and supplemented by reasonable tolerance control, the proposed design structure remains feasible for fabrication, although challenges in physical realization still persist.

In summary, the results of this set of simulation experiments indicate that when the gold film thickness is 0.05 μm, the best TS is achieved, reaching a maximum of 3.86 nm/°C with an R^2^ of 0.9989, demonstrating excellent TS performance.

### 2.5. Cancer Cell Detection

Cancer is a disease characterized by abnormal and uncontrolled cell growth, with diverse types, high heterogeneity, and complex classification. In this study, six common cancer cell types are selected as the research subjects, including cervical cancer (HeLa), skin cancer (Basal), two types of breast cancer (MDA-MB-231 and MCF-7), blood cancer (Jurkat), and adrenal cancer (PC12) [33]. It should be noted that, at the current simulation design stage of photonic crystal fiber biosensors for cancer cell detection, assigning a fixed refractive index value to different cell types is a widely adopted and common practice, largely due to the hazardous nature of cancer cells and the constraints imposed by laboratory safety conditions. The use of fixed refractive index values also facilitates the exploratory investigation of performance enhancement during the sensor design phase. In this study, the reported refractive index values for the above six cancer cell types are adopted, with the normal and cancer cells covering a refractive index range of 1.36–1.401, as detailed in Table 5 [34]. However, the refractive index of real biological cells exhibits non-negligible individual variations and dynamic fluctuations due to a variety of factors, including hydration state, cellular composition, tumor heterogeneity, and the microenvironment. Therefore, during physical fabrication and testing, it is essential to strictly control and ensure that the analyte is not significantly altered by external factors. Under absolutely safe laboratory conditions, direct refractive index measurement of the cell samples under test can also be performed to obtain their actual refractive index values, thereby moving beyond the reliance on fixed refractive indices in numerical simulations and further enhancing the practical application value of the proposed sensing structure. It is worth noting that when using these data to optimize the structural parameters, the variation in RI is too large and uneven, causing an excessive red shift in the loss peak. This shifts the peak to an overly high wavelength range, making it difficult to capture. In contrast, the RI variation induced by temperature changes is relatively moderate. Since both mechanisms ultimately involve changes in RI, the TS is used to determine the optimal sensing structural parameters, which are then employed for subsequent cancer cell detection. The TS performance is continuously improved only during the determination of the optimal structural parameters. In the simulation experiments for cancer cell detection, the changes in refractive index are entirely induced by the difference between normal and cancer cells. Therefore, the temperature can be regarded as constant, and, consequently, no cross-sensitivity issue with temperature arises. For practical experiments, the constant-temperature condition at this stage can be ensured by placing the analyte in an incubator or a more precise temperature control device. During the adjustment of the incubator temperature, the temperature may initially be unstable. A platinum resistance electronic thermometer can be used to monitor the incubator temperature, and the constant-temperature condition is considered to be achieved when the readings of the thermometer and the incubator coincide, thereby avoiding the influence of temperature fluctuations. This is experimentally feasible in practice. In the subsequent simulation experiments, the conducted cancer cell detection and the corresponding performance indicators are all based on the refractive index changes induced by cellular pathological changes.

**Figure 12:**
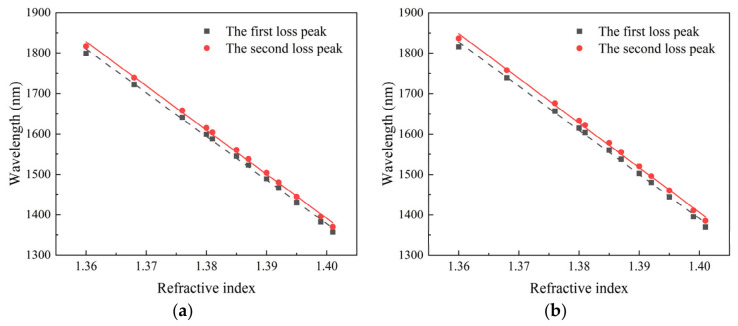
The relationship between loss peaks and changes in refractive index: (**a**) X-polarization; (**b**) Y-polarization.

It can be observed from the table that the RI of cancer cells is higher than that of normal cells. By exploiting this difference in optical properties, the six different cell samples under test are used to replace the anhydrous ethanol liquid in the fiber core for detection. Using the structure with the optimal parameters obtained from the aforementioned three sets of simulation experiments, and by analyzing the performance indicators of normal and cancerous cells, highly sensitive and label-free detection of cancer cells can be achieved. However, a key prerequisite for a high-sensitivity sensing structure is a linear response. A linear fit based solely on the loss peak data corresponding to the RI of a single type of cancer cell does not meet this requirement. Therefore, the loss peaks corresponding to all RI data in Table 5 were linearly fitted, as shown in Figure 12.

At this point, the wavelength sensitivity is determined by the ratio of the shift in peak wavelength Δ*λ_peak_* to the refractive index change Δ*n* [35], as shown in Equation (3). The resulting average sensitivity and R^2^ are presented in Table 6.

(3) $$S_{\lambda }=\frac{\Delta \lambda_{peak}}{\Delta n}\;(\mathrm{nm}/\mathrm{RIU})$$

**Figure 13:**
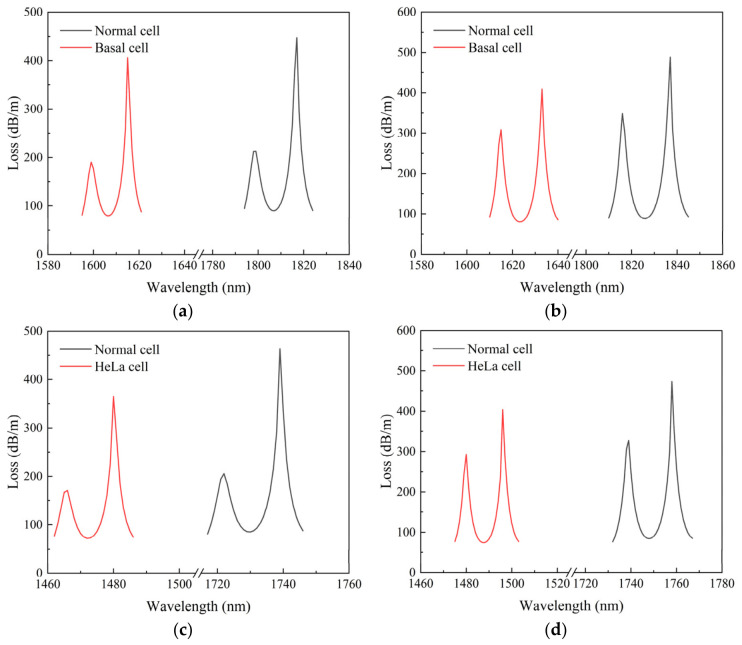

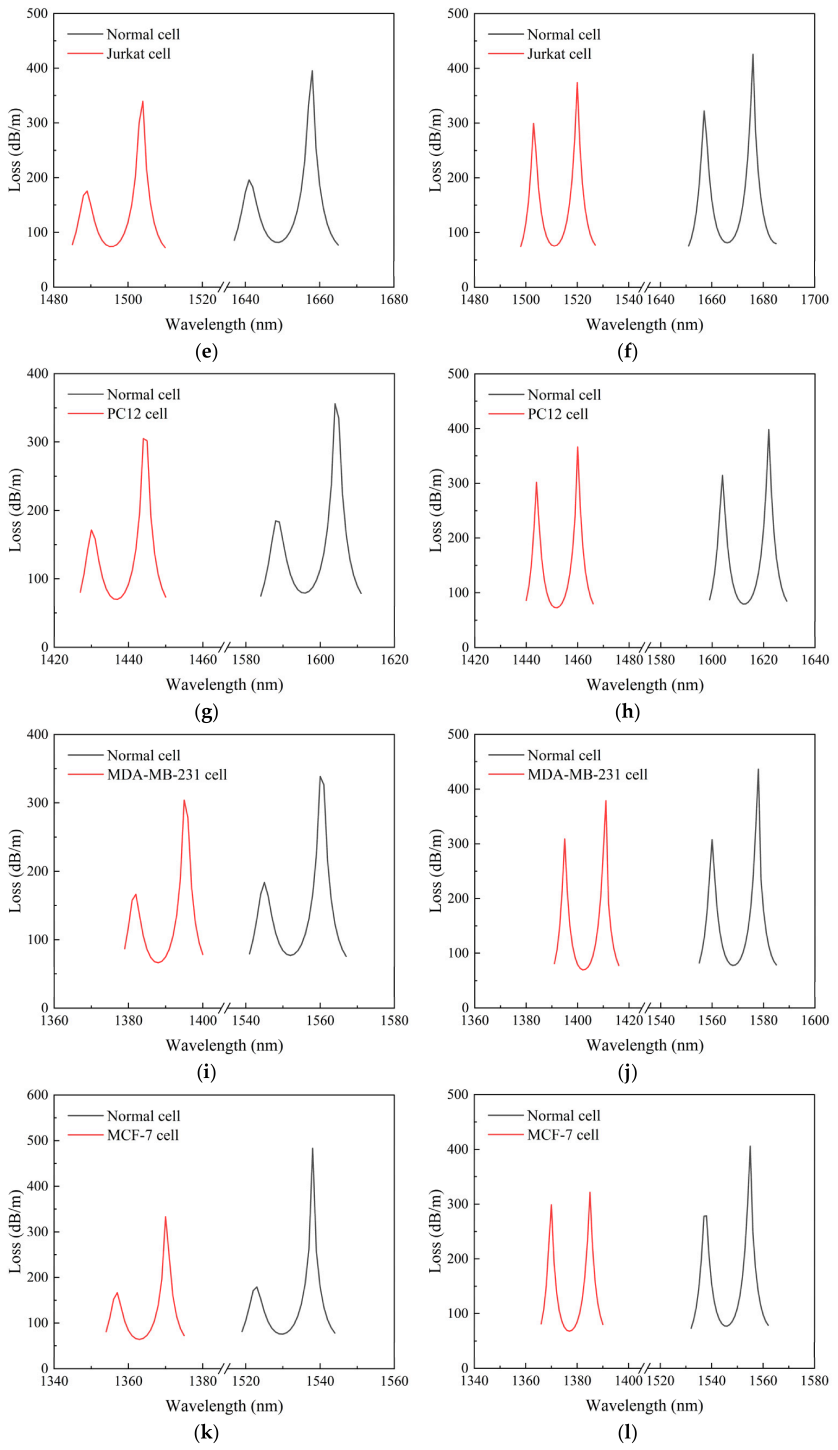
Loss curves of normal cells and cancer cells: (**a**) Basal X-polarization; (**b**) Basal Y-polarization; (**c**) HeLa X-polarization; (**d**) HeLa Y-polarization; (**e**) Jurkat X-polarization; (**f**) Jurkat Y-polarization; (**g**) PC12 X-polarization; (**h**) PC12 Y-polarization; (**i**) MDA-MB-231 X-polarization; (**j**) MDA-MB-231 Y-polarization; (**k**) MCF-7 X-polarization; (**l**) MCF-7 Y-polarization.

From the fitting results, the maximum average sensitivity reaches 11,056.22 nm/RIU, with excellent linear response characteristics. This facilitates the accurate detection of refractive index changes with reliable stability, providing the structure with excellent conditions and support for the subsequent simulated detection experiments on the six types of cancer cells. It is worth noting that during actual measurements, if the refractive index of the analyte changes due to environmental factors during detection, the performance of the sensing structure can still be ensured after the refractive index variation, owing to its linear response characteristic within the refractive index range of 1.36–1.401. Figure 13 illustrates the variation in loss peaks for the six common types of cancer cells and normal cells with a step size of 1 nm.

**Figure 14:**
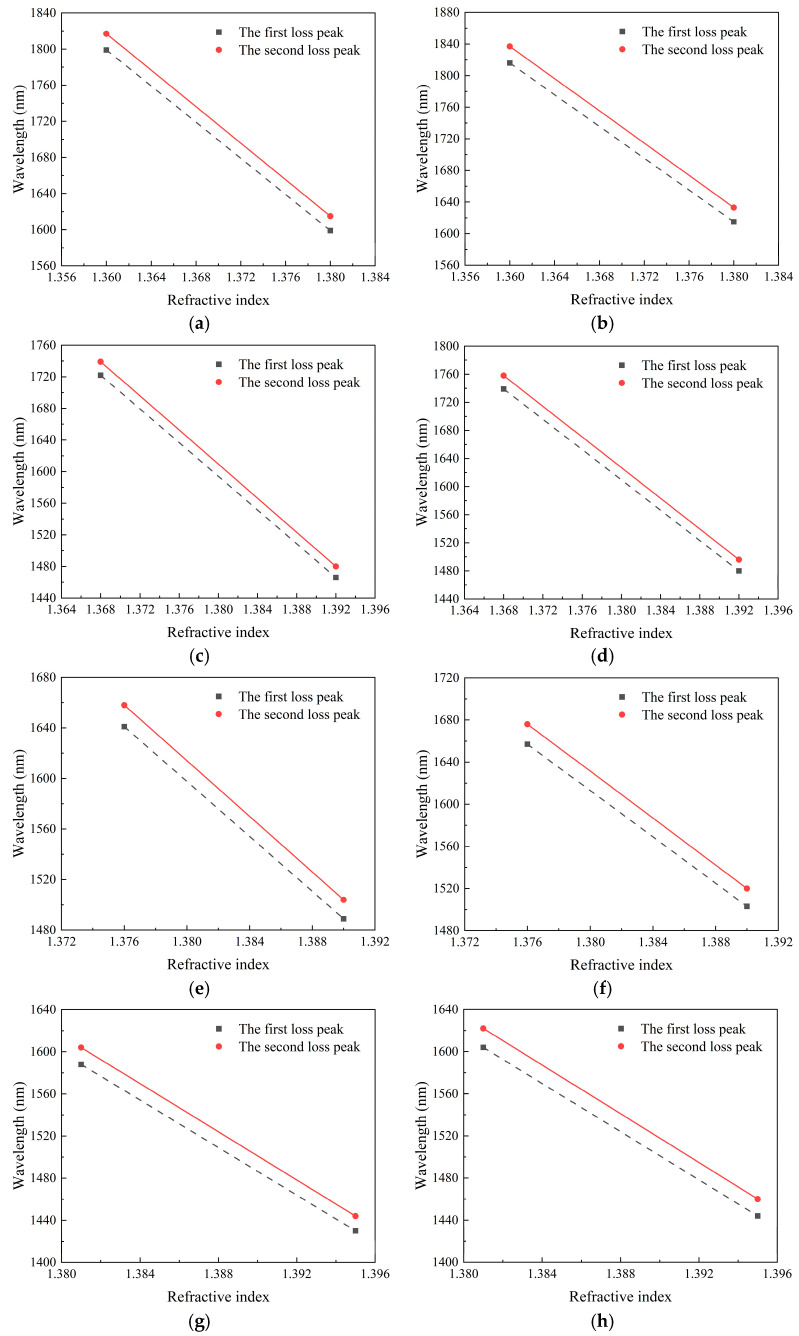

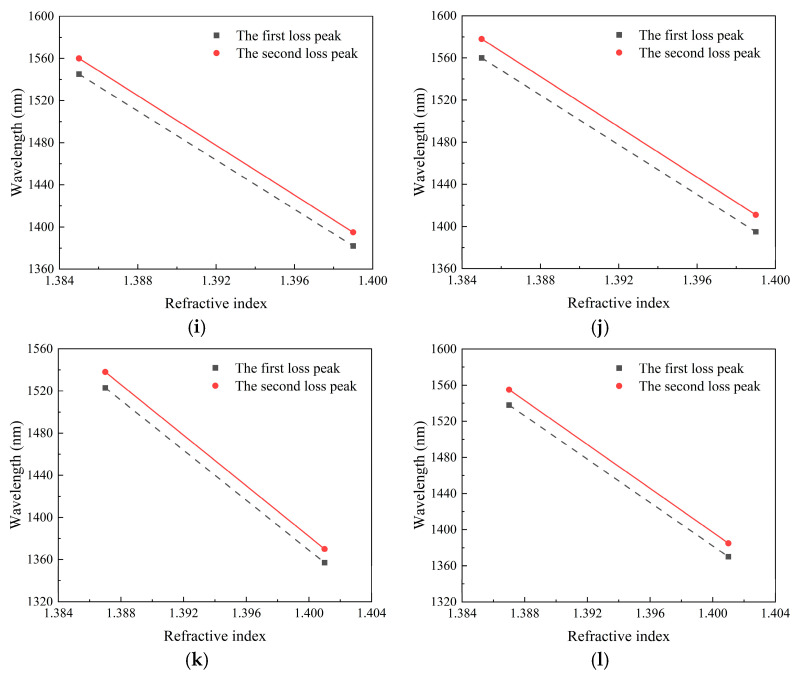
Linear fit curves: (**a**) Basal X-polarization; (**b**) Basal Y-polarization; (**c**) HeLa X-polarization; (**d**) HeLa Y-polarization; (**e**) Jurkat X-polarization; (**f**) Jurkat Y-polarization; (**g**) PC12 X-polarization; (**h**) PC12 Y-polarization; (**i**) MDA-MB-231 X-polarization; (**j**) MDA-MB-231 Y-polarization; (**k**) MCF-7 X-polarization; (**l**) MCF-7 Y-polarization.

Due to the linear response characteristic of this high-sensitivity sensing structure, the loss peaks of six types of normal cells and their corresponding cancer cells were measured separately, as shown in Figure 14. Meanwhile, the corresponding WS were calculated, as presented in Table 7.

It can be seen from the table that the proposed sensing structure exhibits excellent cancer cell detection performance. The WS for the detection of all six types of cancer cells exceed 10,000 nm/RIU, with the highest reaching 12,142.86 nm/RIU, providing a powerful solution for accurate, efficient, and convenient cancer cell detection.

## 3. Results

In the aforementioned simulation experiments on temperature and cancer cell detection, although sensitivity serves as the ultimate indicator for evaluating sensing performance, it is still necessary to select relevant data for comprehensive performance assessment. A prerequisite for the proposed sensing structure to achieve high-precision performance is an excellent resolution (R) [36], which is determined by Equations (4) and (5). Signal-to-noise ratio (SNR) and limit of detection (LOD) are important performance metrics. SNR reflects the clarity and reliability of the sensor signal [37] and is determined by Equation (6), while LOD refers to the smallest change that the sensing structure can detect [38] and is determined by Equation (7). The figure of merit (FOM) is also a key indicator for evaluating sensing performance, determined by Equations (8) and (9). It reflects the sensitivity of the sensing structure to signal changes and the balance between signal and noise. A high FOM value indicates that the sensing structure possesses high sensitivity and signal accuracy, along with a good signal-to-noise ratio, enabling effective noise suppression [39].

(4) $$R_{T}=\frac{\Delta T\times \Delta \lambda_{min}}{\Delta \lambda_{peak}}\;({}^{\circ}C)$$

(5) $$R_{\lambda }=\frac{\Delta n\times \Delta \lambda_{min}}{\Delta \lambda_{peak}}\;(\mathrm{RIU})$$

(6) $$SNR=\frac{\Delta \lambda_{peak}}{FWHM}$$

(7) $$LOD=\frac{FWHM}{1.5\times {\left(SNR\right)}^{0.25}}\;(\mathrm{nm})$$

(8) $$FOM=\frac{S_{T}}{FWHM}\;({}^{\circ}C^{-1})$$

(9) $$FOM=\frac{S_{\lambda }}{FWHM}\;({\mathrm{RIU}}^{-1})$$

Among them, Δ*λ_min_* is a key metric in spectroscopy, and this value is typically set to 0.1 nm. FWHM stands for full width at half maximum.

Performance evaluation was conducted using the optimal TS under the optimal structural parameters, specifically using the parameters of an elliptical curvature of 26:48, a cladding tube thickness of 1.0 μm, a gold film thickness of 0.05 μm, and a TS of 3.86 nm/°C. The relevant data were calculated, and the results are presented in Table 8. Here, the RW refers to the peak value of the loss peak.

It can be observed that the proposed sensing structure achieves a minimum FWHM of 4 nm, an optimal R of 2.27 × 10^−2^ °C, a maximum SNR of 5.5, a best LOD of 1.74 nm, and a maximum FOM of 1.1 °C^−1^, demonstrating excellent temperature performance metrics and great potential in temperature detection. A comparison of temperature performance between the proposed sensing structure and other optical fiber structures based on the SPR effect is presented in Table 9, further confirming the superior temperature detection capability of the proposed structure.

Next, the optimal wavelength sensitivity for cancer cell detection under the optimal structural parameters was selected for performance evaluation. The calculated results of various metrics are presented in Table 10. Here, Δ*λ* represents the peak wavelength shift between normal cells and their corresponding cancer cells for the six types of cancer.

It can be observed that the proposed sensing structure achieves a minimum FWHM of 4 nm. For MCF-7 cell detection, it achieves an optimal WS of 12,142.86 nm/RIU, an optimal R of 8.24 × 10^−6^ RIU, and a maximum FOM of 3035.72 RIU^−1^. For HeLa cell detection, it achieves a maximum SNR of 65.50 and a best LOD of 0.94 nm. Overall, in the simulated detection of the six different types of cancer cells, the wavelength sensitivities all exceed 10,000 nm/RIU, the resolutions all reach the order of 10^−6^, and all other performance indicators exhibit outstanding results, demonstrating balanced and excellent performance. This indicates that the proposed structure holds significant potential for promising applications in the field of cancer cell detection. A comparison of the performance indicators between the designed sensing structure and other optical fiber structures also based on the SPR effect is presented in Table 11, further validating the superior performance of the proposed structure. Should a certain degree of tolerance still occur during fabrication, reference can be made to the optimal sensitivity data obtained from the comparative simulation experiments with different cladding tube and gold film thicknesses, which were optimized based on temperature sensitivity in this study. It can be calculated that when the maximum error reaches 10%, the maximum variation in sensitivity is approximately 8.81%. Even when the simulated detection of cancer cells is performed under the condition of the greatest sensitivity variation, the resulting performance after accounting for the error remains excellent and still holds a considerable advantage over comparable studies.

It is worth noting that when the SPP mode is excited at the interface between the cladding tube of the “egg-shaped” composite structure and the gold film, the SPP causes a sharp increase in the confinement loss, resulting in the appearance of an energy loss peak at a specific wavelength. While the strong SPR effect delivers high sensitivity performance, it also excites an intense energy loss peak, which is typically accompanied by a broadening of the resonance width and thus a larger FWHM. According to Equations (6) and (7), when the shift in the loss peak Δ*λ_peak_* remains unchanged, the LOD is proportional to the 1.25th power of the FWHM, which inevitably degrades the LOD of the sensing structure. Thus, high sensitivity performance paradoxically limits an excellent LOD. Similarly, as can be seen from Equations (3) and (5), the resolution is inversely proportional to the sensitivity. The larger FWHM caused by high sensitivity further restricts the improvement in resolution, which, to a certain extent, affects the practical resolution in real-world spectral interrogation systems. Although sensitivity performance is mutually constrained with resolution and LOD, in this study the designed sensitivity performance, while remaining excellent, still allows the resolution and LOD to hold significant advantages over the comparable studies listed in Table 11.

In this study, the designed sensing structure possesses excellent performance indicators, reflecting the performance potential of the sensor design. Although the silica material in the cladding tubes has a very small thermal expansion coefficient, offering favorable temperature stability, and both the model structure and the fabrication tolerances exhibit a certain degree of feasibility during the experimental fabrication of the fiber, the actual performance during fabrication and testing may still be affected by factors such as light source stability, coupling efficiency, environmental perturbations, and the detection limit of instrumental resolution. In future experiments, further and more comprehensive evaluations should be conducted with respect to the practical instrumental resolution limit and broader robustness issues, so as to verify the actual detection capability of the sensing structure under real working conditions.

## 4. Conclusions

This study proposes and designs a novel anti-resonant fiber sensing structure based on a composite “egg-shaped” configuration. The innovative “egg-shaped” structure is realized by designing a cladding tube consisting of a semicircle and a semi-ellipse, with its inner surface coated with a gold film, while the fiber core is filled with ethanol. Since the refractive index variation induced by temperature changes is relatively small and exhibits a stable trend, temperature sensitivity is employed to determine the optimal structural parameters through three sets of simulation experiments that vary the elliptical aspect ratio, cladding tube thickness, and gold film thickness. The sensing structure with the optimal parameters was applied to the simulated analysis of cancer cell detection, aiming to provide a design reference for the field of high-sensitivity biosensing and to offer feasibility value for applications in cancer cell detection. Six common types of cancer cells were selected in the simulation experiments, namely HeLa cells, Basal cells, MDA-MB-231 cells, MCF-7 cells, Jurkat cells, and PC12 cells. The simulation results show that with an elliptical curvature of 26:48, a cladding tube thickness of 1.0 μm, and a gold film thickness of 0.05 μm, the highest and optimal temperature sensitivity of 3.86 nm/°C is achieved, with a corresponding R^2^ of 0.9989. For the detection of MCF-7 cells, the highest WS, optimal R, and highest FOM are achieved, reaching 12,142.86 nm/RIU, 8.24 × 10^−6^, and 3035.72 RIU^−1^, respectively. For the detection of HeLa cells, the highest SNR and best LOD are obtained, reaching 65.50 and 0.94 nm, respectively. When detecting the six different types of cancer cells, the average WS reaches as high as 11,056.22 nm/RIU, with an R^2^ of 0.9979. Among the six types of cancer cells, the WS exceeds 10,000 nm/RIU for all types, the R reaches the order of 10^−6^ RIU, and the other performance metrics are balanced and excellent. Moreover, owing to its unique detection mechanism, the proposed sensing structure exhibits label-free characteristics, demonstrating balanced and outstanding overall performance. The designed simulated sensing structure has demonstrated excellent comprehensive performance in both temperature and cancer cell detection, showing important application prospects in temperature monitoring and cancer cell detection, and exhibiting great potential for the design and application of high-sensitivity optical fiber sensing in the fields of environmental monitoring and medical prevention and treatment.

## Figures and Tables

**Table 1:** Refractive index of anhydrous ethanol at different temperatures.

Temperature (°C)	Refractive Index
20	1.3605
25	1.3585
30	1.3564
35	1.3546
40	1.3522

**Table 2:** Sensitivity of different ellipse curvatures.

Curvature of Ellipse	Sensitivity of X-Polarization at Different Loss Peaks (nm/°C)		Sensitivity of Y-Polarization at Different Loss Peaks (nm/°C)	
26:48	3.42	3.5	3.5	3.52
26:50	3.46	3.46	3.46	3.52
26:52	3.42	3.46	3.46	3.52

**Table 3:** Sensitivity of different cladding tube wall thicknesses.

Thickness (μm)	Sensitivity of X-Polarization at Different Loss Peaks (nm/°C)		Sensitivity of Y-Polarization at Different Loss Peaks (nm/°C)	
0.8	3.1	3.16	3.14	3.16
0.9	3.42	3.5	3.5	3.52
1.0	3.76	3.76	3.8	3.86

**Table 4:** Sensitivity of different coating thicknesses.

Thickness (μm)	Sensitivity of X-Polarization at Different Loss Peaks (nm/°C)		Sensitivity of Y-Polarization at Different Loss Peaks (nm/°C)	
0.05	3.76	3.8	3.8	3.86
0.1	3.76	3.76	3.8	3.86
0.15	3.74	3.8	3.76	3.86

**Table 5:** Refractive index of different cells.

Type of Cancer	Cell Name	RI of Normal Cell	RI of Cancerous Cell
Skin	Basal cell	1.36	1.38
Cervical	HeLa	1.368	1.392
Blood	Jurkat	1.376	1.39
Adrenal glands	PC12	1.381	1.395
Breast	MDA-MB-231	1.385	1.399
Breast	MCF-7	1.387	1.401

**Table 6:** The average sensitivity of different fitting curves.

Direction	The First Loss Peak		The Second Loss Peak	
	Average Sensitivity (nm/RIU)	R^2^	Average Sensitivity (nm/RIU)	R^2^
X-polarization	10,812.69	0.9980	10,939.87	0.9979
Y-polarization	10,923.33	0.9978	11,056.22	0.9979

**Table 7:** The wavelength sensitivity of different fitting curves.

Type of Cells	Sensitivity of X-Polarization at Different Loss Peaks (nm/RIU)		Sensitivity of Y-Polarization at Different Loss Peaks (nm/RIU)	
Skin cancer (Basal)	10,000.00	10,100.00	10,050.00	10,200.00
Cervical cancer (HeLa)	10,666.67	10,791.67	10,791.67	10,916.67
Blood cancer (Jurkat)	10,857.14	11,000.00	11,000.00	11,142.86
Adrenal glands cancer (PC12)	11,428.57	11,285.71	11,428.57	11,571.43
Breast cancer (MDA-MB-231)	11,642.86	11,785.71	11,785.71	11,928.57
Breast Cancer (MCF-7)	11,857.14	12,000.00	12,000.00	12,142.86

**Table 8:** Performance metrics that increase with temperatures.

T (°C)	RW (nm)	CL (dB/m)	FWHM (nm)	S (nm/°C)	R (°C)	SNR	LOD (nm)	FOM (°C^−1^)
20	1832	464.68	5	3.8	2.63 × 10^−2^	3.8	2.39	0.76
25	1851	462.83	5	4	2.50 × 10^−2^	4.0	2.36	0.8
30	1871	493.38	5	3.4	2.94 × 10^−2^	3.4	2.45	0.68
35	1888	524.46	4	4.4	2.27 × 10^−2^	5.5	1.74	1.1
40	1910	522.03	4	NA	NA	NA	NA	NA

**Table 9:** Performance comparison of TS with some previous work.

References	Temperature Sensitivity
[11]	37.9 pm/°C
[12]	2.932 nm/°C
[13]	2.59 nm/°C
[14]	0.713 nm/°C
[15]	1.6107 nm/°C
[16]	2.86 nm/°C
This work	3.86 nm/°C

**Table 10:** Performance metrics for cancer cell detection.

Type of Cells	Δλ (nm)	CL (dB/m)	FWHM (nm)	S (nm/RIU)	R (RIU)	SNR	LOD (nm)	FOM (RIU^−1^)
Basal	204	408.90	5	10,200.00	9.80 × 10^−6^	40.80	1.32	2040.00
HeLa	262	403.81	4	10,916.67	9.16 × 10^−6^	65.50	0.94	2729.17
Jurkat	156	373.57	5	11,142.86	8.97 × 10^−6^	31.20	1.41	2228.57
PC12	162	366.40	4	11,571.43	8.64 × 10^−6^	40.50	1.06	2892.86
MDA-MB-231	167	378.41	4	11,928.57	8.38 × 10^−6^	41.75	1.05	2982.14
MCF-7	170	321.49	4	12,142.86	8.24 × 10^−6^	42.50	1.04	3035.72

**Table 11:** Performance comparison of WS with some previous work.

References	Type of Cells	S (nm/RIU)	R (RIU)	LOD (nm)	FOM (RIU^−1^)
[17]	Basal	2500.00	4.00 × 10^−5^	NA	NA
	HeLa	2916.66	3.42 × 10^−5^	NA	NA
	Jurkat	3571.42	2.80 × 10^−5^	NA	NA
	PC12	3571.42	2.80 × 10^−5^	NA	NA
	MDA-MB-231	4285.71	2.33 × 10^−5^	NA	NA
	MCF-7	4285.71	2.33 × 10^−5^	NA	NA
[18]	HeLa	5500.00	1.82 × 10^−5^	24.00	NA
	Jurkat	6000.00	1.67 × 10^−5^	14.00	NA
	PC12	7571.43	1.32 × 10^−5^	14.00	NA
	MDA-MB-231	9428.57	1.06 × 10^−5^	14.00	NA
	MCF-7	10,714.28	0.93 × 10^−5^	14.00	NA
[19]	Basal	3500.00	2.9 × 10^−5^	NA	78.00
	HeLa	5000.00	2.0 × 10^−5^	NA	91.00
	Jurkat	5714.00	1.8 × 10^−5^	NA	78.00
	PC12	6429.00	1.6 × 10^−5^	NA	60.00
	MDA-MB-231	7143.00	1.4 × 10^−6^	NA	125.00
	MCF-7	7143.00	1.4 × 10^−6^	NA	100.00
[20]	Basal	3000.00	3.33 × 10^−5^	NA	NA
	HeLa	3333.33	3.00 × 10^−5^	NA	NA
	Jurkat	4285.72	2.33 × 10^−5^	NA	NA
	PC12	4285.72	2.33 × 10^−5^	NA	NA
	MDA-MB-231	5714.28	1.75 × 10^−5^	NA	NA
	MCF-7	5714.28	1.75 × 10^−5^	NA	NA
[21]	Basal	3750.00	2.67 × 10^−5^	NA	71.45
	HeLa	5417.00	1.85 × 10^−5^	NA	99.72
	Jurkat	6071.00	1.65 × 10^−5^	NA	117.00
	PC12	7500.00	1.33 × 10^−5^	NA	119.60
	MDA-MB-231	9643.00	1.04 × 10^−5^	NA	79.90
	MCF-7	11,429.00	8.75 × 10^−6^	NA	65.08
[22]	Basal	1500.00	6.70 × 10^−5^	NA	NA
	HeLa	1666.67	6.00 × 10^−5^	NA	NA
	Jurkat	1428.57	7.00 × 10^−5^	NA	NA
	PC12	1428.57	7.00 × 10^−5^	NA	NA
	MDA-MB-231	2142.86	4.67 × 10^−5^	NA	NA
	MCF-7	2142.86	4.67 × 10^−5^	NA	NA
This work	Basal	10,200.00	9.80 × 10^−6^	1.32	2040.00
	HeLa	10,916.67	9.16 × 10^−6^	0.94	2729.17
	Jurkat	11,142.86	8.97 × 10^−6^	1.41	2228.57
	PC12	11,571.43	8.64 × 10^−6^	1.06	2892.86
	MDA-MB-231	11,928.57	8.38 × 10^−6^	1.05	2982.14
	MCF-7	12,142.86	8.24 × 10^−6^	1.04	3035.72

“NA” stands for “Not Available,” indicating that the relevant data were not reported in that reference.

## Data Availability

Data underlying the results presented in this paper are not publicly available at this time but may be obtained from the authors upon reasonable request.
